# Impaired PTEN Expression in T Cells Drives Resistance to Treg-Mediated Immune Regulation in Multiple Sclerosis

**DOI:** 10.3390/cells14181445

**Published:** 2025-09-16

**Authors:** Janine Schlöder, Bettina Trinschek, Felix Luessi, Helmut Jonuleit

**Affiliations:** 1 Department of Dermatology, University Medical Center of the Johannes Gutenberg-University, Langenbeckstr. 1, 55131 Mainz, Germanyjonuleit@uni-mainz.de (H.J.); 2 ActiTrexx GmbH, c/o University Medical Center of the Johannes Gutenberg-University, Langenbeckstr. 1, 55131 Mainz, Germany; 3 Department of Neurology, University Medical Center of the Johannes Gutenberg-University, Langenbeckstr. 1, 55131 Mainz, Germany; luessi@uni-mainz.de

**Keywords:** multiple sclerosis, immune regulation, Treg resistance, PKB hyperactivation, PTEN expression, CD4^+^ T cells, IL-6

## Abstract

The regulation of T cell-mediated immune responses is essential for maintaining immune homeostasis and preventing autoimmune diseases. In multiple sclerosis (MS), impaired immunoregulatory control allows autoreactive T cells to persist, as effector T cells (Teff) display reduced susceptibility to regulatory T cells (Treg). This resistance to Treg-mediated tolerance is linked to altered IL-6 signaling and hyperactivation of protein kinase B (PKB/c-Akt). However, the mechanisms leading to increased PKB phosphorylation remain poorly understood. Here, we examined the expression of phosphatase and tensin homolog PTEN, a key phosphatase that negatively regulates PKB/c-Akt activation. We found that PTEN protein expression rapidly declines in activated Teff from MS patients. To clarify whether PTEN downregulation contributes to Treg resistance, we used PTEN-specific siRNA to modulate PTEN expression in Teff from healthy donors. PTEN knockdown resulted in accelerated IL-6 production, enhanced PKB phosphorylation, and reduced responsiveness to Treg-mediated suppression, similar to Treg resistance observed in MS. This study reports disrupted PTEN expression in activated Teff from MS patients. Our findings highlight that PTEN is critical for effective immune regulation of T cells, and suggest its dysregulation contributes to impaired immune tolerance in MS.

## 1. Introduction

Multiple sclerosis (MS) is a chronic inflammatory, neurodegenerative autoimmune disease of the central nervous system, caused by auto-aggressive immune reactions against myelinated axons [1]. T cells from the peripheral blood of relapsing-remitting MS (RRMS) patients exhibit a reduced responsiveness to the suppressive abilities of regulatory T cells (Treg) [2,3]. This impaired regulation of T cells is generally referred to as Treg resistance and can significantly contribute to the pathogenesis of autoimmune diseases. The state of Treg resistance is reversible: Several studies have shown that T cells are efficiently suppressed again when patients undergo treatment with disease-modifying drugs (DMD) such as dimethyl fumarate or interferon beta-1a [4,5].

In recent years, researchers have turned their attention to identifying molecular signaling pathways linked to Treg resistance in T cells. Among the key players in this process are extracellular factors like IL-6. Elevated levels of IL-6 and its dysregulated production are heavily implicated in the pathogenesis of several chronic autoimmune diseases, including MS, rheumatoid arthritis, Crohn’s disease, and type 1 diabetes [6,7,8,9]. Trinschek et al. revealed that T cells from RRMS patients exhibit accelerated IL-6 production following TCR-mediated activation. This elevated IL-6 production correlated with increased IL-6R expression and a reduced T cell sensitivity to Treg-mediated immune suppression [2]. The binding of IL-6 to its receptor triggers the activation of various intracellular signaling mechanisms, such as the JAK-STAT [10,11] and PI3K-Akt pathways [12,13]. In MS, the activation state of protein kinase B (PKB/c-Akt) plays a crucial role in the development of Treg resistance [14]. Studies of Treg-resistant T cells from untreated RRMS patients demonstrated hyperactivation of PKB/c-Akt. By inhibiting PKB/c-Akt activation, the sensitivity of T cells to Treg-mediated suppression was restored [2]. However, the mechanisms behind PKB/c-Akt hyperactivation in T cells from RRMS patients remain unclear.

Therefore, we focused on analyzing specific proteins that regulate the activation and inactivation of PKB/c-Akt. One important negative regulator of PKB/c-Akt activation is phosphatase and tensin homolog PTEN, which dephosphorylates phosphatidylinositol-3,4,5-trisphosphate (PIP_3_) to phosphatidylinositol-4,5-bisphosphate (PIP_2_), resulting in less PIP_3_ being available for Akt activation and thus inhibiting the Akt signaling pathway [15,16]. The objective of our study was to analyze the function of PTEN in the context of Treg resistance in more detail. For this purpose, we compared the PTEN expression in T cells from untreated MS patients and healthy donors in order to examine whether the observed hyperactivation of PKB/c-Akt is due to impaired PTEN expression and can therefore be associated with impaired immune regulation.

## 2. Materials and Methods

### 2.1. MS Patients and Healthy Controls

We collected blood samples from a total of 31 MS patients in order to investigate the immune regulation of peripheral T cells. Twenty-eight patients suffered from relapse-remitting multiple sclerosis (RRMS), and three patients had a clinically isolated syndrome (CIS). Twenty MS patients showed a relapse; the remaining patients were in remission. Further clinical characteristics are listed in the supplementary part (Table S1). All patients fulfilled the revised McDonald criteria for multiple sclerosis and were considered “untreated”, i.e., they had not received previous treatment or immunosuppressive agents six months before the time point of analysis [17]. PBMC obtained from healthy volunteers were included as controls. All PBMC were isolated by density gradient centrifugation within 16 h after blood collection and immediately applied in functional assays.

### 2.2. Isolation and Culture of Human Peripheral Immune Cells

PBMC from either MS patients or healthy donors were isolated from buffy coats or heparinized syringes within 16 h after blood collection using BioColl density gradient centrifugation (Bio & SELL, Feucht, Germany). Blood samples were kept at room temperature before PBMC enrichment. After isolation, human cells were cultured in X-VIVO-15 (Lonza, Verviers, Belgium). For some experiments, PBMC were pre-incubated with 500 ng/mL recombinant human IL-6 (CellGenix, Freiburg, Germany) for 16 h in a humidified 37 °C/5% CO_2_ incubator.

### 2.3. Flow Cytometry

Flow cytometric analysis was performed using the following antibodies: anti-human CD3 (SK7), anti-human CD4 (SK3), anti-human CD8 (SK1), anti-human CD14 (M5E2), anti-human CD19 (HIB19), and anti-human CD69 (FN50), all from BD Biosciences (Heidelberg, Germany); anti-human CD25 (4E3), anti-human CD127 (MB15-18C9), anti-human CD40L (REA238), and anti-human IL-6 receptor (REA291), all from Miltenyi Biotec (Bergisch Gladbach, Germany). Cell viability during flow cytometric analysis was determined using 7-AAD (Thermo Fisher Scientific, Dreieich, Germany). For surface staining, PBMC or T cells were incubated with the indicated antibodies for 20 min at 4 °C and washed twice with PBS supplemented with 0.5% HSA + 1 mM EDTA + 10 µg/mL Sandoglobulin (CSL Behring, Hattersheim, Germany). To detect phosphorylated PKB/c-Akt or PTEN, cells were fixed at 37 °C with BD Cytofix Buffer for 10 min, then permeabilized with BD Phosflow Perm Buffer III for 30 min on ice, washed twice with BD Stain Buffer, and stained with BD Phosflow anti-Akt pS473 (M89-61) or BD Phosflow anti-PTEN (A2B1) according to manufacturer’s instructions (all buffers and antibodies from BD Biosciences, Heidelberg, Germany). For intracellular staining of Foxp3, cells were fixed and permeabilized using Foxp3 Transcription Factor Fix/Permeabilization Kit (Thermo Fisher Scientific, Dreieich, Germany)) and stained with anti-human Foxp3 (259D, Biolegend, Koblenz, Germany). Stained samples were analyzed on a BD FACSVia cytometer (FACSVia Research Software v.1.1) or alternatively on an LSRII instrument using FACSDiva Software v8.0.2 (BD Biosciences, Heidelberg, Germany).

### 2.4. Isolation of Immune Cell Subsets

Untouched CD4^+^ T cells were isolated using the CD4^+^ T Cell Isolation Kit (Miltenyi Biotec, Bergisch Gladbach, Germany) according to the manufacturer’s instructions. For some experiments, CD4^+^ T cells were enriched by positive selection using anti-CD4 Microbeads (Miltenyi Biotec, Bergisch Gladbach, Germany). Isolated CD4^+^ T cells were then depleted of intrinsic Treg with anti-CD25 Dynabeads (Thermo Fisher Scientific, Dreieich, Germany). The average purity of CD4^+^CD25^−^ T cells was >95%. A portion of the isolated T cells was activated with 0.5 µg/mL anti-human CD3 mAb (OKT-3, Bio X cell, Rüsselsheim, Germany) + 1 µg/mL anti-human CD28 mAb (CD28.2, BD Biosciences, Heidelberg, Germany) for different time periods.

CD4^+^CD25^+^Foxp3^+^ Treg were isolated from PBMC using anti-CD25 Microbeads (Miltenyi Biotec, Bergisch Gladbach, Germany) and depleted of contaminating CD8^+^, CD14^+,^ and CD19^+^ cells with Dynabeads (Thermo Fisher Scientific, Dreieich, Germany) as described previously [18]. Purity was routinely >85%, and Treg functionality was ensured in standard suppressor assays.

For some experiments, PBMC were depleted of CD3^+^ or CD25^+^ cells using corresponding Dynabeads (1 bead/cell; Thermo Fisher Scientific, Dreieich, Germany).

### 2.5. Suppressor Assays

CD4^+^CD25^−^ T cells (10^5^ cells/well, 96-well plate, flat bottom) were stimulated with 0.1 µg/mL anti-human CD3 mAb (OKT-3, Bio X cell, Rüsselsheim, Germany) and cultured in the presence or absence of different Treg ratios. CD3-depleted PBMC (5 × 10^4^ cells/well) were used as a costimulus. For some experiments, CD25-depleted PBMC (10^5^ cells/well) were stimulated with 0.1 µg/mL anti-human CD3 mAb (OKT-3, Bio X cell) and co-cultured with or without different Treg ratios. On day 3, ^3^H-Tdr was added to each well (37 kBq/well) and cells were cultured for an additional 16 h. T cell proliferation was measured by ^3^H-Tdr incorporation using a liquid β-scintillation counter. For blockade experiments, cultures were supplemented with 30 ng/mL anti-IL-6R antibody (Tocilizumab, Roche, Grenzach-Wyhlen, Germany) or 0.1 µM PKB/c-Akt VIII inhibitor (Calbiochem/Merck, Darmstadt, Germany). In each suppression assay, T cells from the test group (e.g., MS patient, IL-6 pre-incubated T cells) were analyzed in direct parallel with T cells from a control group (e.g., healthy donor, untreated), using the same Treg from an independent third healthy donor. The inclusion of control group T cells served both as an internal control to confirm Treg functionality in each assay and as a comparison for the test group.

### 2.6. Cytokine Analysis

Treg-depleted PBMC from MS patients or healthy controls were cultured with or without Treg (ratio 1:1) from an independent third-party healthy donor. Stimulation was performed using anti-human CD3 mAb (OKT-3, Bio X cell, Rüsselsheim, Germany). After 72 h, cytokine concentration in the supernatants was quantified by Cytometric Bead Array (BD Bioscience, Heidelberg, Germany) according to the manufacturer’s protocol and evaluated using GraphPad Prism 6 (Statcon, Witzenhausen, Germany).

### 2.7. RNA Isolation, cDNA Synthesis, and qRT-PCR

RNA was extracted from 1–2 × 10^6^ CD4^+^ T cells using peqGOLD Micro RNA Kit according to the manufacturer’s instructions (VWR, Darmstadt, Germany). cDNA was synthesized from 100 ng of the isolated RNA by reverse transcription with iScript™ cDNA Synthesis Kit (Bio-Rad Laboratories, Feldkirchen, Germany) and the supplied random hexamer primers. All quantitative RT-PCR reactions were performed in triplicate on a Rotor-Gene Q cycler using QuantiTect SYBR Green PCR Kit (Qiagen, Hilden, Germany). EEF1A, PTEN, and IL-6 mRNA expression levels were measured with the appropriate QuantiTect Primer Assay according to the manufacturer’s protocol (Qiagen, Hilden, Germany). The mRNA levels of the housekeeping gene EEF1A were used for normalization, and relative expression levels were calculated with the 2^−ΔΔCT^ method.

### 2.8. PTEN Knockdown Experiments

For a successful knockdown of the PTEN protein, the PTEN GeneSolution siRNA Kit (Qiagen, Hilden, Germany) was used, including the following siRNAs:

Hs_PTEN_6           Target sequence: AAGGCGTATACAGGAACAATA

Hs_PTEN_9           Target sequence: TCGACTTAGACTTGACCTATA

Hs_PTEN_8           Target sequence: ATCGATAGCATTTGCAGTATA

Hs_PTEN_4           Target sequence: TCGGCTTCTCCTGAAAGGGAA

AllStars Negative Control siRNA (Qiagen, Hilden, Germany) was employed as a scrambled control. Negatively isolated CD4^+^CD25^−^ T cells were transfected with AMAXA^®^ Human T cell Nucleofector Kit according to the manufacturer’s protocol (Lonza, Verviers, Belgium). Briefly, 5 × 10^6^ T cells were resuspended in 100 µL Nucleofector Solution (per sample) supplemented with 1 µM PTEN siRNA mix or negative control siRNA. Nucleofector program U-014 was used to efficiently transfect T cells. After nucleofection, T cells were cultured overnight in X-VIVO-15 in a humidified 37 °C/5% CO_2_ incubator, and knockdown efficiency was analyzed at the indicated time points using qRT-PCR (Bio-Rad Laboratories, Feldkirchen, Germany) and Western blot (Thermo Fisher Scientific, Dreieich, Germany).

### 2.9. SDS-PAGE and Western Blotting

Cell pellets were lysed in Cell Extraction supplemented with 1 mM PMSF (Thermo Fisher Scientific, Dreieich, Germany) and Protease Inhibitor Cocktail (Sigma-Aldrich, Steinheim, Germany) for 30 min on ice with vortexing at 10 min intervals. Total protein concentration of cell lysates was determined with Micro BCA Protein Assay Kit (Thermo Fisher Scientific, Dreieich, Germany) according to the manufacturer’s protocol. Protein separation was performed using the NuPAGE Bis-Tris electrophoresis system (all NuPAGE reagents from Thermo Fisher Scientific, Dreieich, Germany). Briefly, up to 15 µg of total protein were mixed with NuPAGE LDS Sample Buffer and NuPAGE Reducing Agent and loaded onto NuPAGE 4–12% (gradient) Bis-Tris gels (denaturing conditions, 200 V, 60 min, with NuPAGE MOPS SDS Running Buffer). The separated proteins were transferred onto PVDF membranes (0.45 µm pore size, Life Technologies, Darmstadt, Germany) using the semi-wet XCell II Blotting system (30 V, 60 min, Thermo Fisher Scientific, Dreieich, Germany). For immunoblot analysis, membranes were blocked in TBST buffer/5% BSA for at least 1 h at room temperature. The incubation with primary antibodies was carried out according to the manufacturer’s recommendations. For primary incubation, the following antibodies were used: rabbit anti-PTEN mAb (138G6), rabbit anti-β-Actin mAb (13E5), rabbit anti-pan-Akt mAb (C67E7), all from Cell Signaling Technology (Frankfurt, Germany), mouse anti-PKB/Akt pSer437 mAb (11E6, Nanotools, Teningen, Germany), and mouse anti-β-Actin mAb (8226, Abcam, Cambridge, United Kingdom). Purified goat anti-rabbit IgG antibody (Cell Signaling Technology, Frankfurt, Germany) and rabbit anti-mouse IgG antibody (Abcam, Cambridge, United Kingdom) conjugated to horseradish peroxidase were employed for chemiluminescent detection with Amersham ECL Prime Western Blotting Detection Reagent (GE Healthcare Life Sciences, Freiburg, Germany). Chemiluminescence was documented using the ChemiDoc Imaging System (Bio-Rad Laboratories, Feldkirchen, Germany). Western blots were analyzed and quantified with ImageJ software v.1.53.

### 2.10. Statistical Analysis

Data are shown as mean values ± SEM or SD. Group comparisons were carried out using unpaired Student’s *t*-tests against the respective control (as indicated). A *p*-value of less than 0.05 was considered statistically significant and is indicated in the corresponding figures:

*: *p* < 0.05

**: *p* < 0.01

***: *p* < 0.001

****: *p* < 0.0001

n.s.: not significant

## 3. Results

### 3.1. IL-6 and PKB/c-Akt Activity Contribute Directly to Treg Resistance in MS

**Figure 1:**
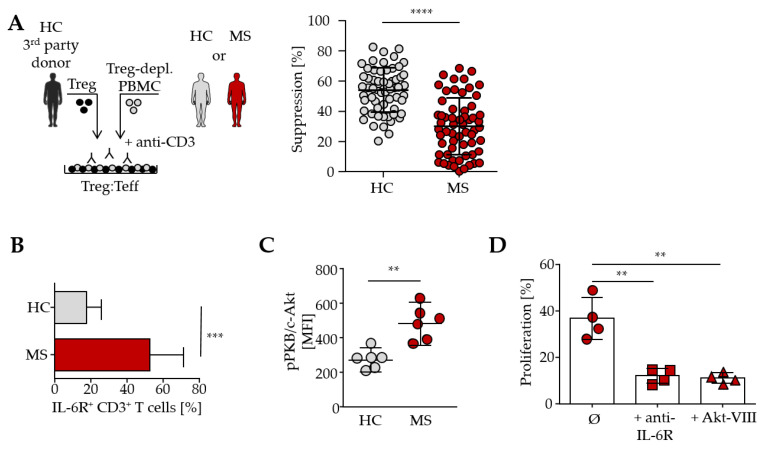
Treg resistance in MS is linked to IL-6 and the PKB signaling pathway. (**A**) Treg-depleted PBMC from untreated MS patients (red) or HC (gray) were co-cultured with Treg from a 3rd-party healthy donor (black) and stimulated with anti-CD3 mAb. T cell proliferation was determined by ^3^H-Tdr incorporation on day three. Plot shows suppression ± SD in the presence of Treg (PBMC–Treg ratio 4:1) normalized to proliferation of T cells alone, *p*-value of T cells from MS patients relative to T cell proliferation of HC is shown (****: *p* < 0.0001). Each dot represents one individual donor (*n* = 60). Combined data from previously published and newly generated experiments [2,5]. (**B**) IL-6R expression within CD3^+^ T cells of PBMC from HC or MS was determined by flow cytometry. Bars show mean IL-6R expression ± SD of *n* = 10 individuals. *p*-value relative to CD3^+^ T cells from HC is shown (***: *p* < 0.001). (**C**) PKB/c-Akt phosphorylation was determined by flow cytometry within CD3^+^ T cells of MS or HC. Six different experiments with *p*-value relative to MFI of HC are shown (**: *p* < 0.01). (**D**) CD4^+^CD25^−^ T cells from MS patients were cocultured with Treg from a healthy donor and stimulated with anti-CD3 mAb alone (circle) or in the presence of anti-IL-6R mAb (quadrats) or Akt-VIII inhibitor (triangles). CD3-depleted PBMC served as a costimulus. Each dot represents the percentage of proliferation in the presence of Treg, normalized to T cells alone (*n* = 4); *p*-values relative to untreated MS T cells are shown (**: *p* < 0.01).

In recent years, we have studied the blood of MS patients of various ages regarding T cell-mediated immune regulation. All MS patients included in our studies were considered therapy-naïve or “untreated”, i.e., they did not receive any immunosuppressive treatment six months before blood donation and were clinically stable. Whether T cells are responsive to Treg-mediated suppression was analyzed using an established in vitro assay. Treg-depleted PBMC of either MS or healthy controls (HC) were cocultured with isolated Treg from independent third-party healthy donors (Figure 1A). Since Treg are functionally disturbed in MS patients, we determined T cell function in the absence of patient-intrinsic Treg [19].

As previously demonstrated by us and others, T cells from RRMS patients show a reduced susceptibility towards Treg-mediated immunosuppression, herein termed Treg resistance. Cumulative data of 60 patients indicate an average suppression rate of 54.3 ± 14.6% for Treg-sensitive HC T cells and 30.4 ± 18.9% for Treg-resistant MS T cells (Cohen’s *d* = 1.42, 95%-CI [1.02, 1.82]) at a PBMC–Treg ratio of 4:1 (Figure 1A). The cumulative analysis presented here integrates both previously published and newly acquired data on T cells from MS patients, allowing for a more comprehensive evaluation of Treg resistance [2,5].

In addition, Treg-resistant T cells are characterized by an increased IL-6R expression and enhanced phosphorylation of PKB/c-Akt. By blocking the IL-6 signaling pathway or inhibiting PKB/c-Akt activity, the sensitivity of T cells to Treg-mediated suppression can be fully restored (Figure 1B–D). Surprisingly, we did not observe any significant differences in the expression of activation markers or cytokine production in T cells from MS and HC (Figures S1 and S2).

In line with our previous studies, Treg resistance was consistently observed in Teff from untreated/therapy-naïve MS patients, whereas Teff from patients under active therapy displayed restored Treg sensitivity [4,5]. Notably, in these earlier studies, we did not detect correlations of Treg resistance with age, sex, or clinical disease activity, but rather with treatment status alone. Based on this robust observation, the present study was designed to further characterize the underlying mechanisms of Treg resistance.

### 3.2. Altered PTEN Expression in T Cells from MS Patients After Activation

**Figure 2:**
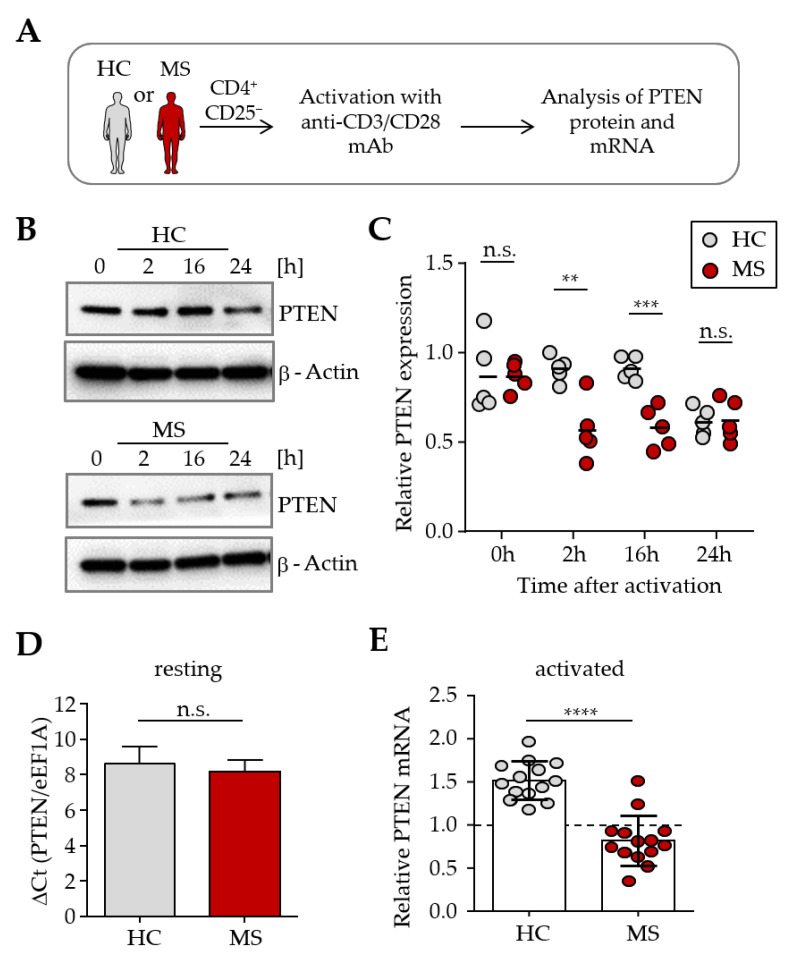
Accelerated decrease in PTEN expression in MS T cells after activation. (**A**) Experimental setup: CD4^+^CD25^−^ T cells were isolated from peripheral blood samples of healthy donors (HC, gray) and MS patients (MS, red), followed by polyclonal activation with 0.5 µg/mL anti-CD3 mAb and 1 µg/mL anti-CD28 mAb. PTEN expression was examined at both the RNA and protein levels at defined time points. (**B**) Western blot depicts total PTEN protein expression of T cells at different time points before and after polyclonal activation. β-Actin was used as a loading control. (**C**) Densitometric analysis of PTEN protein expression in resting as well as activated T cells. PTEN expression was normalized to the corresponding β-Actin control at the indicated time points. Each dot represents PTEN expression of one individual donor (*n* = 5), *p*-values of PTEN expression in MS T cells relative to HC T cells are shown (**: *p* < 0.01, ***: *p* < 0.001, n.s.: not significant). (**D**) Relative PTEN mRNA expression in resting T cells from HC and MS patients. Bars show mean PTEN mRNA expression ΔCt (PTEN/eEF1A) ± SD of *n* = 14 individuals (n.s.: not significant). (**E**) Relative PTEN mRNA expression two hours after activation with 0.5 µg/mL anti-CD3 mAb and 1 µg/mL anti-CD28 mAb. PTEN expression in activated T cells was normalized to PTEN expression in the corresponding unstimulated control. Each dot represents one individual donor (*n* = 14, Cohen’s *d* = 2.71, 95% CI [1.66, 3.75], ****: *p* < 0.0001).

In follow-up experiments, we carried out comparative studies on the expression of phosphatase PTEN in T cells from untreated MS patients that had been validated as Treg-resistant in suppression assays. We compared them to Treg-sensitive T cells from healthy donors. For this purpose, CD4^+^CD25^−^ T cells were isolated from the peripheral blood and polyclonally activated for 2, 16, and 24 h with anti-CD3 and anti-CD28 mAb. T cells were harvested at the respective time points after activation, and the extracted proteins were subsequently analyzed by Western blot (Figure 2A). In the resting state, T cells from MS patients and healthy donors showed similar PTEN expression. After polyclonal activation, a faster decrease in PTEN protein expression was observed in T cells from MS patients compared to HCs. CD4^+^ T cells from healthy controls showed a marked reduction in PTEN expression 24 h after activation, whereas T cells from MS patients exhibited a significant decrease as early as 2 h post-activation (Figure 2B,C). In parallel, PTEN mRNA expression levels in CD4^+^ T cells before and after polyclonal activation were determined by qPCR. Elongation factor 1A (eukaryotic translation elongation factor 1A, eEF1A) was used as a housekeeping gene. The results showed comparable PTEN mRNA expression levels in resting T cells from MS patients and healthy donors. Two hours after polyclonal activation, T cells from healthy donors displayed a clear upregulation of PTEN mRNA, which was not observed in T cells from untreated MS patients (Figure 2D,E).

### 3.3. PTEN Knockdown in T Cells from Healthy Donors Reduces Sensitivity to Immunoregulation

Our previous studies have shown an altered PTEN expression pattern in T cells from MS patients after TCR-mediated activation. To analyze the impact of impaired PTEN expression on the suppressibility of T cells, PTEN knockdown experiments were performed with T cells from the peripheral blood of healthy donors. We hypothesized that a reduction in PTEN expression could lead to hyperactivation of PKB/c-Akt and consequently to decreased T cell responsiveness to Treg-mediated suppression.

**Figure 3:**
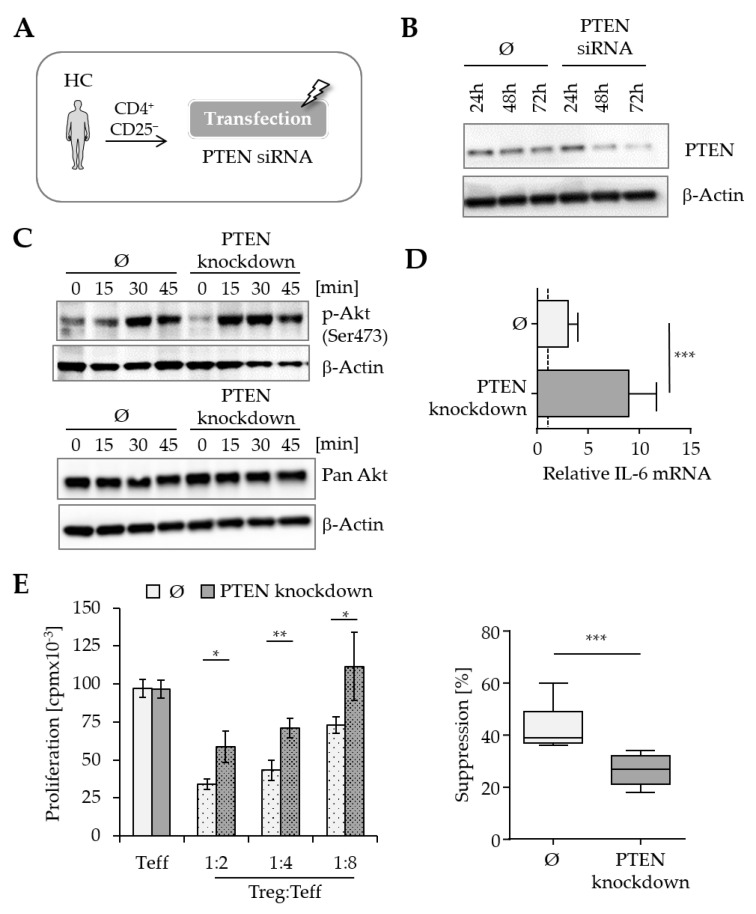
PTEN knockdown induces Treg resistance in HC T cells by modulating PKB/c-Akt activity and IL-6 production. (**A**) Experimental setup for transfection of T cells: Untouched CD4^+^CD25^−^ T cells, isolated from healthy donors (HC), were treated with PTEN-specific siRNA or scrambled control siRNA using the AMAXA^®^ device. (**B**) Analysis of PTEN protein expression after transfection: Western blot depicts PTEN expression of T cells at different time points after transfection with control siRNA (Ø) and PTEN-specific siRNA. β-Actin was used as a loading control. (**C**) Transfected T cells were activated with 0.5 µ/mL anti-CD3 mAb and 1 µg/mL anti-CD28 mAb for 0, 15, 30, and 45 min, and the phosphorylation of PKB/c-Akt was analyzed by Western blot. β-Actin served as a loading control. (**D**) Transfected T cells were stimulated with 0.5 µg/mL anti-CD3 mAb and 1 µg/mL anti-CD28 mAb for 4 h, and IL-6 mRNA expression was assessed by qRT-PCR. Unstimulated T cells served as a control. To evaluate the change in IL-6 mRNA expression upon activation, the expression levels in activated T cells were normalized to those of the corresponding unstimulated control. The bar graphs show the mean ± SD from *n* = 5 independent experiments (***: *p* < 0.001). (**E**) Transfected T cells were activated with 0.1 µg/mL anti-CD3 mAb in the presence or absence of freshly isolated Treg. CD3-depleted PBMC were used as a costimulus. Proliferation of T cells was measured via ^3^H-Tdr incorporation. Left: Graph shows suppression of T cells in one representative experiment (*: *p* < 0.05, **: *p* < 0.01). Right: Box plots show percentage of suppression in the presence of Treg (T cell–Treg ratio 4:1) of *n* = 9 experiments, Ø = scrambled control siRNA, ***: *p* < 0.001.

In order to investigate this hypothesis, freshly isolated CD4^+^CD25^−^ T cells from healthy donors were transfected with PTEN-specific siRNA. A successful reduction in PTEN expression was demonstrated at both the mRNA and protein levels at different time points after transfection (Table S2, Figure S3 and Figure 3A,B). Since phosphatase PTEN is a key antagonist of Akt activation, we examined the phosphorylation levels of PKB/c-Akt in T cells with PTEN knockdown in subsequent experiments. Following TCR-mediated stimulation, these cells exhibited increased phosphorylation of PKB/c-Akt (Ser473). The basal expression of PKB remained unaffected (Figure 3C). Additionally, comparative analyses of IL-6 production revealed an accelerated increase in IL-6 mRNA levels in T cells with PTEN knockdown (Figure 3D). Further in vitro studies clarified the impact of reduced PTEN expression on T cell suppression. Transfected T cells were cocultured with freshly isolated Treg from an independent healthy donor following polyclonal stimulation. The proliferation of T cells after TCR-mediated activation was unaffected by the reduction in PTEN expression. In the absence of Treg, the proliferation rate of T cells from both the control and the PTEN knockdown group was comparable. Interestingly, in the presence of Treg, T cells with PTEN knockdown exhibited a significantly decreased responsiveness to immunosuppression by Treg. Cumulative data from nine independent experiments revealed an average T cell suppression of 43.3 ± 3.2% in the control groups and 26.3 ± 2.1% in the PTEN knockdown groups (Cohen’s *d* = 2.34, 95% CI [0.94, 3.75]) (Figure 3E).

Reduction in PTEN expression in T cells from healthy donors led to both enhanced PKB/c-Akt activation and increased production of IL-6 mRNA. As a consequence, the regulability of these T cells was significantly diminished, mimicking the Treg-resistant phenotype observed in MS patients. These results highlight a critical role for the phosphatase PTEN in maintaining T cell responsiveness to Treg-mediated suppression. The findings further suggest that the increased PKB/c-Akt activity seen in T cells from MS patients may result from an impaired or dysregulated downregulation of PTEN expression.

### 3.4. IL-6 Impairs PTEN Expression and Promotes Treg Resistance in Effector T Cells

Given the critical role of the proinflammatory cytokine IL-6 in the development of Treg resistance, we investigated its impact on the expression of the phosphatase PTEN [2,3]. These experiments aimed to elucidate potential mechanisms underlying the accelerated decline in PTEN expression observed in T cells from MS patients.

**Figure 4:**
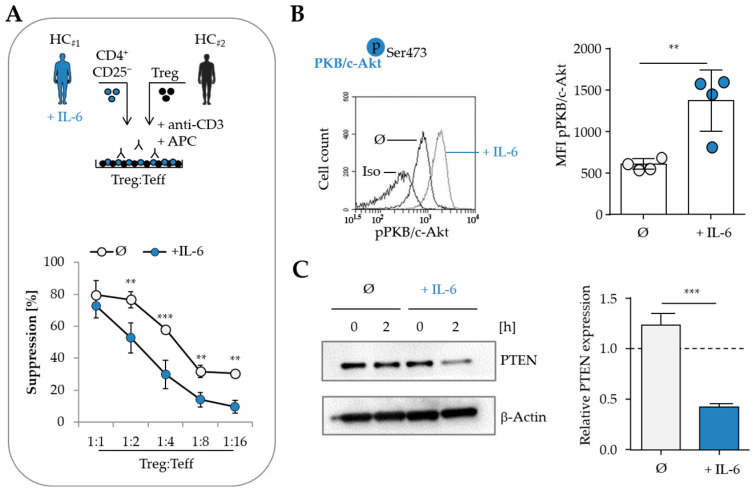
IL-6 promotes Treg resistance by enhancing PKB/c-Akt phosphorylation and suppressing PTEN in activated T cells. (**A**) PBMC from HC were cultured overnight in the absence (gray) or presence (blue) of 500 ng/mL IL-6. Afterwards, CD4^+^CD25^−^ T cells were isolated and activated with 0.1 µg/mL anti-CD3 mAb together with different ratios of freshly isolated Treg from a second healthy donor. CD3-depleted PBMC (hereafter referred to as APC) served as a costimulus. T cell proliferation was determined by ^3^H-Tdr incorporation on day three and displayed as mean ± SD of triplicate measurements, *n* = 4 (**: *p* < 0.01, ***: *p* < 0.001). (**B**) Phosphorylation of PKB/c-Akt in T cells was assessed by flow cytometry following overnight culture with (blue) or without IL-6 (gray). Data are presented as mean fluorescence intensity (MFI) of pPKB/c-Akt (*n* = 4, **: *p* < 0.01). (**C**) Isolated T cells were activated with 0.5 µg/mL anti-CD3 and 1 µg/mL anti-CD28 mAb for two hours; unstimulated T cells served as controls. Left: Total proteins were isolated, and PTEN protein expression was analyzed via Western blot. Right: Densitometric analysis of PTEN protein expression in activated CD4^+^ T cells. The expression of PTEN in each individual was first normalized to β-Actin and then to its respective unstimulated control. Bars show mean PTEN protein expression of *n* = 3 individual experiments (***: *p* < 0.001).

PBMC from healthy donors were incubated with high concentrations of IL-6 for at least 16 h at 37 °C. Subsequently, CD4^+^CD25^−^ T cells were isolated and assessed for their responsiveness to Treg-mediated suppression in an in vitro assay. In line with previous findings from our group, pre-treatment with IL-6 markedly reduced the suppressibility of Teff (Figure 4A) [2]. While the proliferation of untreated T cells was efficiently suppressed by functional Treg, IL-6-treated T cells exhibited pronounced resistance to suppression. This reduced responsiveness correlated with the phosphorylation status of PKB/c-Akt. IL-6-treated T cells displayed increased phosphorylation of PKB/c-Akt at Ser473 compared to untreated cells (Figure 4B). Further analyses revealed that IL-6 also affected the expression of PTEN. In the resting state, PTEN expression was comparable between control and IL-6-treated T cells. However, upon TCR stimulation, a substantial reduction in PTEN expression was observed in IL-6-treated cells (Figure 4C).

Collectively, these findings demonstrate a direct effect of IL-6 on PTEN expression. IL-6 treatment leads to enhanced downregulation of PTEN following TCR-mediated activation, resulting in hyperactivation of the PKB/c-Akt signaling pathway and reduced susceptibility of Teff to Treg-mediated suppression.

## 4. Discussion

In the present study, we investigated the functional role of the phosphatase and tensin homolog PTEN in the regulation of T cell immune responses. Our results provide evidence that not only the mere presence of PTEN but likely also the kinetics of its regulation following T cell activation determines T cell susceptibility to Treg-mediated suppression.

The molecular mechanisms underlying the loss of Treg-mediated tolerance in patients with autoimmune diseases are still incompletely understood. Our analyses demonstrate that common T cell activation markers are expressed to the same extent in both MS patients and healthy controls. This is consistent with the findings of Schneider et al., who furthermore observed no differences in the proportion of memory T cells in Treg-resistant MS patients compared to Treg-sensitive healthy controls [3]. Similar results were obtained regarding the production of T cell-associated cytokines. Treg-resistant T cells from MS patients produce a cytokine milieu comparable to that of Treg-sensitive T cells from healthy donors. This observation has been confirmed both by our group and by Schneider’s research team [3]. These findings indicate that Treg resistance is driven by alterations occurring at deeper intracellular or molecular levels.

The sensitivity of T cells to the suppressive functions of Treg may depend, at least in part, on the activation status of protein kinase B (PKB/c-Akt) [14]. Previous studies by Wehrens et al. demonstrated increased phosphorylation of PKB/c-Akt in T cells from patients with juvenile idiopathic arthritis [20]. This hyperactivation correlated with a markedly reduced susceptibility of T cells to Treg-mediated suppression. Comparable findings have been reported in MS, where Treg-resistant T cells from the peripheral blood of patients also exhibit elevated PKB/c-Akt phosphorylation levels [2,3]. Thus, PKB/c-Akt activation and its regulation represent a central molecular hub in the emergence of Treg resistance in autoimmune diseases [21]. PTEN acts as a critical negative regulator of PKB/c-Akt signaling [15]. In our study, reduced PTEN expression was detected in activated T cells from MS patients, suggesting it may represent a key driver of PKB/c-Akt hyperactivation and the resulting Treg resistance. In addition to PKB/c-Akt itself, PTEN thus emerges as another pivotal factor essential for effective immune regulation of T cells. Functionally, PTEN counteracts phosphoinositide 3-kinase activity and serves as a major checkpoint in the control of PKB/c-Akt signaling [22].

Several studies have linked impaired PTEN expression or function to the pathogenesis of various diseases. In particular, numerous malignancies are associated with loss-of-function mutations in the PTEN gene, leading to constitutive activation of the PI3K/Akt pathway and increased tumor cell proliferation [23,24,25]. Moreover, mouse models with T cell-specific PTEN deficiency have demonstrated that uncontrolled Akt activation significantly contributes to the development of autoimmune disorders and lymphomas [26,27]. Recent studies by Kortam et al. report a downregulation of the PTEN gene expression in the serum of 100 MS patients compared to healthy controls, accompanied by an upregulation of the AKT gene expression [28]. Notably, PTEN expression was significantly lower in patients with more severe neurological impairment (EDSS ≥ 3.5) than in those with milder symptoms (EDSS < 3.5), suggesting a potential correlation between PTEN levels and disease severity. The findings of our study further support a correlation between dysregulated PTEN expression and autoimmunity. Specifically, CD4^+^CD25^−^ T cells from untreated MS patients displayed a significantly accelerated decline in PTEN expression following polyclonal stimulation compared to T cells from healthy donors. This altered kinetic pattern was selectively observed in Treg-resistant CD4^+^CD25^−^ T cells derived from MS patients. We hypothesize that the accelerated loss of PTEN contributes to a hyperactivated phenotype characterized by enhanced cytokine production and a more rapid T cell response in the context of MS. This idea is supported by previous studies showing that effector T cells from MS patients produce IL-6 more rapidly and resist Treg-mediated suppression due to sustained Akt activation [2]. Moreover, hyperactivation of transcription factors such as NFAT1 and increased T-B cell interactions further amplify T cell responsiveness in MS, indicating that altered activation kinetics are a hallmark of the disease [29,30].

PTEN also plays a fundamental role in T cell immune regulation. For instance, Lee et al. demonstrated that overexpression of PTEN in mice suffering from arthritis markedly ameliorated clinical symptoms, coinciding with reduced frequencies of inflammatory Th17 cells and increased Treg populations [31]. Similarly, Suzuki et al. reported that CD4^+^ T cells isolated from mice with T cell-specific PTEN deletion exhibited a predominantly autoreactive phenotype, increased proliferative capacity, and enhanced secretion of inflammatory Th1 cytokines [32]. These T cells also showed hyperactivation of PKB/c-Akt signaling. Consistent with these findings, targeted PTEN knockdown in CD4^+^CD25^−^ T cells from healthy donors, as performed in our study, resulted in enhanced PKB/c-Akt activation and accelerated production of the proinflammatory cytokine IL-6. Additional in vitro analyses revealed that these PTEN-deficient T cells exhibited a decreased susceptibility to Treg-mediated suppression, similar to the Treg-resistant phenotype of T cells observed in untreated MS patients. Interestingly, the overall proliferative capacity of T cells was not affected by PTEN knockdown. Thus, targeted modulation of PTEN expression can influence T cell function as well as their regulation by Treg [33].

Given the strong association between impaired PTEN expression and disease development, elucidating the factors contributing to dysregulated PTEN levels is of critical importance. PTEN expression is regulated by multiple mechanisms, ranging from extracellular factors such as cytokines to non-coding RNA [34]. Among these, miR-21 has emerged as a prominent negative regulator of PTEN expression. Several studies have reported elevated miR-21 levels in the cerebrospinal fluid and peripheral T cell populations of MS patients [35,36]. Further studies by Lindberg et al. identified miR-17-5p as an additional regulator of PTEN expression in the context of T cell activation. In CD4^+^ T cells from MS patients, miR-17-5p was found to be upregulated and associated with a corresponding downregulation of its target genes, including PTEN, upon stimulation with anti-CD3/CD28 monoclonal antibodies [37]. In addition, extracellular factors such as TGF-β, which is significantly elevated in the serum of MS patients, have been shown to suppress PTEN expression [38,39,40].

In MS, impaired responsiveness of T cells to Treg-mediated suppression has been associated with accelerated IL-6 mRNA production and hyperactivation of PKB/c-Akt [2,3]. Interestingly, a comparable phenotype can be induced in T cells from healthy donors. When PBMC from healthy individuals are pre-incubated with high amounts of IL-6, the resulting T cells exhibit reduced susceptibility to Treg-mediated suppression compared to untreated controls. This experiment suggests that Treg resistance may not be inherently disease-specific but rather dependent on the inflammatory cytokine milieu. Furthermore, our experiments demonstrated that IL-6 pre-treatment modulates PTEN expression upon subsequent T cell activation in a manner analogous to the alterations observed in MS. The precise mechanism by which IL-6 regulates PTEN remains unclear. However, previous studies have shown that IL-6 activates the transcription factor STAT3, which in turn promotes the expression of miR-21 [41]. MiR-21 has been shown to directly inhibit PTEN. Both STAT3 and miR-21 have been implicated in MS pathogenesis. Schneider et al. reported elevated levels of phosphorylated STAT3 in Treg-resistant T cells from MS patients, while studies by Munoz et al. demonstrated increased miR-21 expression [3,35]. These findings strongly support the hypothesis that IL-6-induced downregulation of PTEN in pre-treated T cells from healthy donors may be mediated through the STAT3/miR-21 axis. However, further experiments are required to directly test this pathway in the context of MS, for example, by selectively modulating STAT3 activity or miR-21 expression in patient-derived T cells. Such analyses would provide a deeper mechanistic understanding of how PTEN is controlled and may reveal additional nodes for therapeutic intervention.

The identification of PTEN as a potential contributor to the development of Treg resistance offers important insights into the underlying pathological mechanisms of autoimmune diseases. A detailed understanding of such mechanisms is essential for identifying novel therapeutic targets and expanding the range of more effective intervention strategies. Although PTEN plays a pivotal role in regulating T cell activation through the PI3K/AKT signaling pathway, its ubiquitous expression across tissues and cell types, as well as pleiotropic functions, limit its suitability as a direct therapeutic target. Pharmacological modulation of PTEN is further complicated by the risk of systemic side effects, including impaired tissue homeostasis, immune dysregulation, and tumorigenesis, so that only targeted approaches (e.g., localized or cell-specific inhibition) are possible [42,43]. In our study, we observed that PTEN is rapidly downregulated upon activation in T cells from MS patients, suggesting that restoring or enhancing PTEN activity could be beneficial in counteracting Treg resistance. Strategies to increase PTEN function have been explored in oncology, including PTEN protein delivery, inhibition of PTEN-targeting miRNA such as miR-21 using antisense oligonucleotides, and exogenous PTEN gene introduction via viral vectors [44,45,46]. While these approaches demonstrate a proof of concept, their translation to MS would require cell-type-specific targeting, ideally focusing on Teff to minimize systemic side effects. Indeed, systemic increases in PTEN activity could carry significant risks, including broad immune dysregulation or immunosuppression, disruption of metabolic homeostasis [47], as well as adverse effects on neural development and synaptic function, such as microcephaly, decreased synaptic density, and neuronal dysfunction [48,49]. Future studies should therefore aim to develop strategies for selective PTEN modulation in T cells (e.g., antibody-directed nanoparticle delivery), balancing therapeutic efficacy with safety considerations.

Nevertheless, PTEN may hold substantial promise as a valuable biomarker. Its expression pattern, particularly when assessed in combination with other molecular parameters such as PKB/c-Akt activation status, could help predict patient responsiveness to Treg-based therapies, for example. Interestingly, disease-modifying drugs (DMDs) such as dimethyl fumarate (DMF) and interferon-beta (IFN-β) have been shown to restore Treg responsiveness in T cells from MS patients by normalizing PKB/c-Akt activation [4,5]. Given this, it is reasonable to hypothesize that PTEN expression, as a negative regulator of PKB/c-Akt, undergoes a parallel normalization. To directly address this possibility, longitudinal analyses of MS patients before and at defined time points after initiation of disease-modifying therapy will be essential. Such paired assessments would not only provide mechanistic insight into the role of PTEN but also establish direct clinical relevance.

In the context of autoimmune diseases such as MS, it is likely that successful therapeutic strategies will require a combination approach to achieve long-lasting immune tolerance [50,51]. One possible strategy could involve first restoring the susceptibility of autoreactive T cells to Treg-mediated suppression using DMD, followed by expanding and enhancing Treg function through CD4-mediated activation to strengthen regulatory control [52]. In addition, PTEN and PKB/c-Akt could serve as dynamic biomarkers. By tracking the normalization kinetics of these proteins following the initiation of disease-modifying therapy, clinicians could identify the optimal timing for secondary interventions, such as pharmacological Treg activation methods. Furthermore, long-term stabilization of tolerance could be supported by monitoring biomarkers, such as PTEN expression patterns, PKB activation status, and additional immune signatures, to guide individualized adjustments of therapy, thereby minimizing the risk of relapse and adverse effects.

## 5. Conclusions

In summary, our results provide strong evidence that PTEN is essential for an efficient immune regulation of T cells. Modulation of PTEN expression leads to significant changes in PKB/c-Akt activation and the IL-6 signaling pathway, thus affecting T cell responsiveness towards Treg-mediated suppression.

## Data Availability

The original contributions presented in this study are included in the article/Supplementary Material. Further inquiries can be directed to the corresponding author(s).

## Supplementary Materials

The following supporting information can be downloaded at: https://www.mdpi.com/article/10.3390/cells14181445/s1. Table S1: Clinical characteristics of MS patients; Table S2: Efficiency of PTEN knockdown in CD4^+^ T cells from healthy donors; Figure S1: Comparative analysis of activation markers on T cells from healthy donors versus MS patients; Figure S2: Comparative analysis of cytokine production of T cells from healthy donors versus MS patients; Figure S3: Flow cytometric analysis of PTEN knockdown.

## Abbreviations

The following abbreviations are used in this manuscript:

APC	Antigen-presenting cell
CIS	Clinically isolated syndrome
DMD	Disease-modifying drug
DMF	Dimethyl fumarate
EDSS	Expanded disability status scale
eEF1A	Eukaryotic translation elongation factor 1 A
GFP	Green fluorescent protein
HC	Healthy control
^3^H-Tdr	^3^H-thymidine
mAb	Monoclonal antibody
MFI	Mean fluorescent intensity
miR	Micro RNA
mRNA	Messenger RNA
MS	Multiple sclerosis
PBMC	Peripheral blood mononuclear cell
PBS	Phosphate-buffered saline
PCR	Polymerase chain reaction
PIP_2_	Phosphatidylinositol-4,5-bisphosphate
PIP_3_	Phosphatidylinositol (3,4,5)-trisphosphate
PKB	Protein kinase B
PMSF	Phenylmethylsulfonyl fluoride
PTEN	Phosphatase and Tensin homolog
qRT-PCR	Quantitative real-time PCR
RNA	Ribonucleic acid
RRMS	Relapsing-remitting multiple sclerosis
SD	Standard deviation
SEM	Standard error of the mean
siRNA	Small interfering RNA
STAT	Signal transducers and activators of transcription
TCR	T cell receptor
Teff	Effector T cell
Treg	Regulatory T cell
