# Supplementary material for: Impaired PTEN Expression in T Cells Drives Resistance to Treg-Mediated Immune Regulation in Multiple Sclerosis
Source: Cells. 2025 Sep 16;14(18):1445. doi: 10.3390/cells14181445 (PMC12468420; doi:10.3390/cells14181445)
Supplement: Supplementary file 1 [file cells-14-01445-s001.zip › cells-3837037-supplementary.pdf]

Table S1: Clinical characteristics of MS patients.

Peripheral blood mononuclear cells were collected in heparinized tubes from 31 MS patients (age 18 to 68 years). Twenty-eight patients suffered from relapse-remitting multiple sclerosis (RRMS) and three patients had a clinically isolated syndrome (CIS). Twenty MS patients showed a relapse, the remaining patients were in remission. At the time point of analysis, all MS patients were considered "untreated", i.e. they did not receive any immunosuppressive treatment six months before blood donation and were clinically stable. The Expanded Disability Status Scale (EDSS) was used to quantify disability (0-7.5).

No.	Sex	Age (yr)	Disease course	Disease duration (yr)	Level of disability (EDSS)	State
1.	F	20	RRMS	0.13	0	Remission
2.	F	18	RRMS	2.16	2	Relapse
3.	F	25	RRMS	0	2	Relapse
4.	F	31	RRMS	8.39	7.5	Remission
5.	F	28	RRMS	0.11	1.5	Relapse
6.	F	21	RRMS	0	2	Relapse
7.	F	55	RRMS	2.69	3	Remission
8.	F	37	RRMS	0.78	0	Remission
9.	F	36	RRMS	0.1	2	Remission
10.	F	30	RRMS	4.57	1	Remission
11.	F	52	RRMS	9.43	1	Relapse
12.	F	34	RRMS	10.16	1	Remission
13.	F	40	CIS	2.12	2	Relapse
14.	F	68	RRMS	23.67	2	Remission
15.	F	29	RRMS	0	0	Relapse
16.	M	50	RRMS	0.25	2	Relapse
17.	M	30	RRMS	0.18	0	Remission
18.	M	24	RRMS	1.34	0	Remission
19.	M	25	RRMS	0.2	1	Relapse
20.	M	30	CIS	11.03	0	Remission
21.	M	39	RRMS	0.19	0	Relapse
22.	M	35	CIS	0.06	1	Relapse
23.	M	27	RRMS	1.5	6	Relapse
24.	M	54	RRMS	1.99	2	Relapse
25.	M	36	RRMS	0.08	2	Relapse
26.	M	25	RRMS	0.2	1	Relapse
27.	M	56	RRMS	18.06	5	Relapse
28.	M	29	RRMS	11.08	0.5	Relapse
29.	M	27	RRMS	0.07	1.5	Relapse
30.	M	23	RRMS	0.31	1	Relapse
31.	M	43	RRMS	0.56	2	Relapse

Table S2: Efficiency of PTEN knockdown in $CD4^{+}$ T cells from healthy donors.

Isolated $CD4^{+}CD25^{-}$ T cells from the peripheral blood of healthy donors were transfected with 1 µM PTEN-specific siRNA mix. As a control approach some of the T cells were treated with 2 µg GFP plasmid. 24 hours after electroporation, PTEN knockdown was determined by qRT-PCR. GFP expression of control T cells was analyzed by flow cytometry. The table summarizes the knockdown efficiency of n = 9 independent experiments.

Experiment	#1	#2	#3	#4	#5	#6	#7	#8	#9
Knockdown [%]	74	80	81	67	80	72	69	66	75
GFP^{+} cells [%]	61	70	65	63	69	59	57	63	68

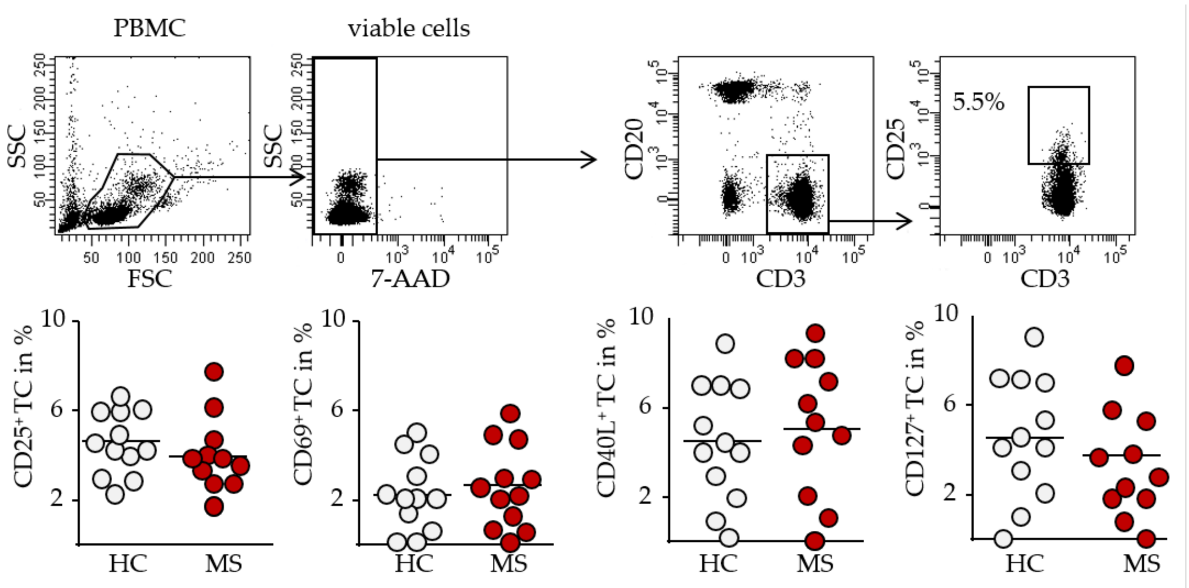

Figure S1: Comparative analysis of activation markers on T cells from healthy donors versus MS patients.

PBMC were isolated from the peripheral blood of healthy donors and untreated RRMS patients and stained with the corresponding antibodies according to the manufacturer's protocol. Subsequently, stained cells were measured on LSRII with FACS Diva Software v8.0.2 (BD Bioscience, Heidelberg, Germany).

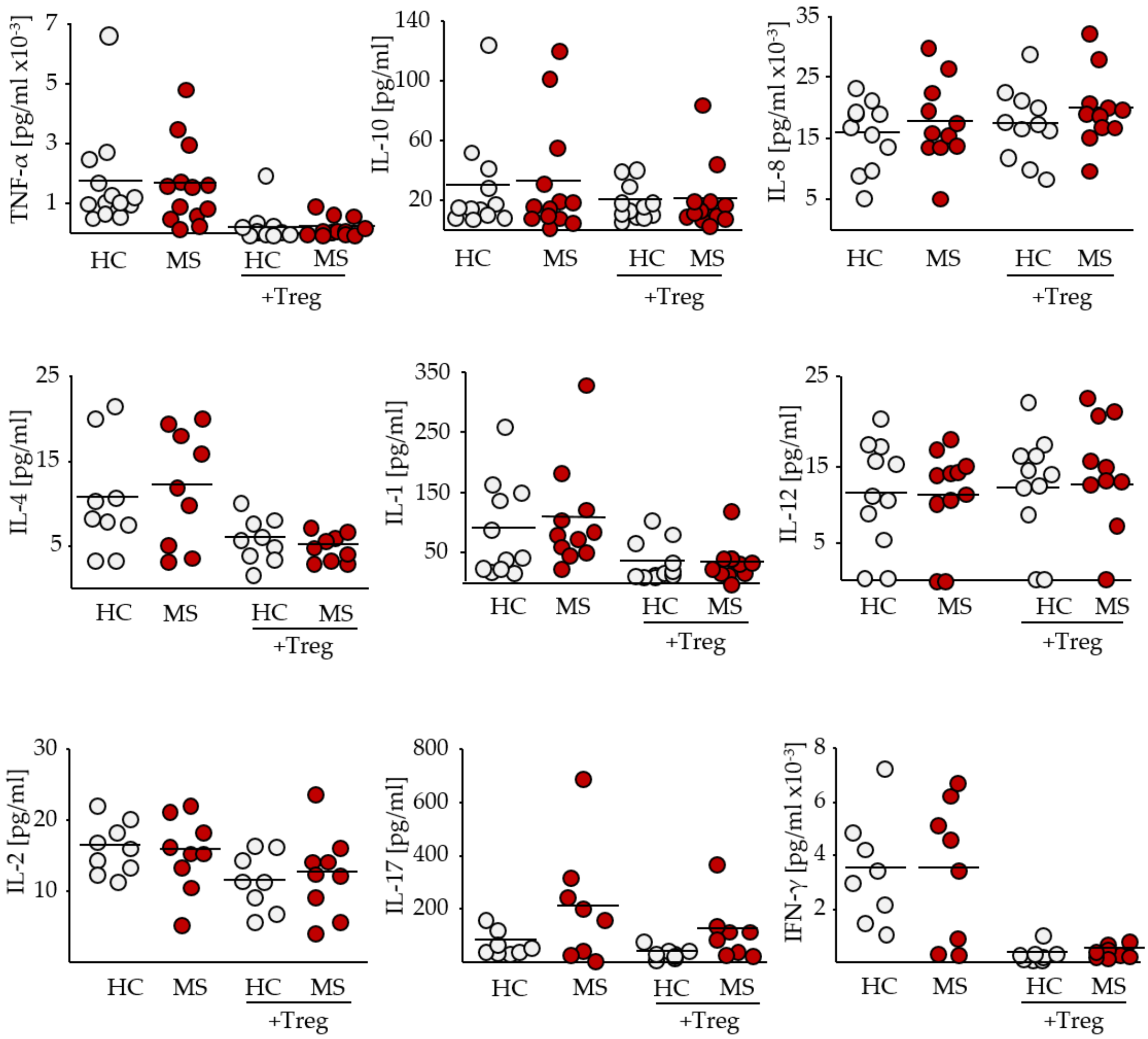

Figure S2: Comparative analysis of cytokine production of T cells from healthy donors versus MS patients.

Treg-depleted PBMC from healthy donors (HC) or MS patients were cultured in presence or absence of Treg (ratio 1:1) and stimulated with anti-CD3 mAb. Supernatants were collected 72 hours after stimulation. Cytometric Bead Array (BD Bioscience, Heidelberg, Germany) was performed following manufacturer's instructions to quantify cytokines in supernatants.

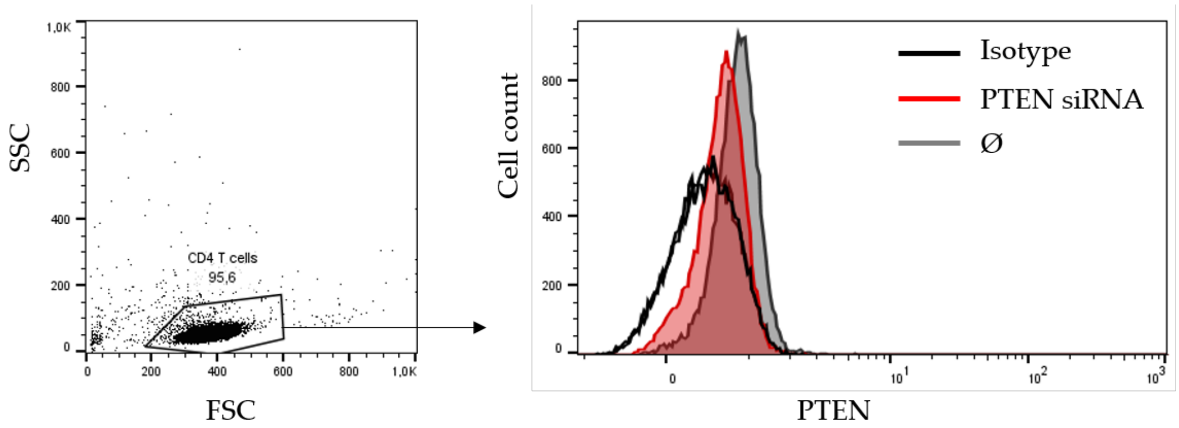

Figure S3: Flow cytometric analysis of PTEN knockdown.
Untouched $CD4^{+}CD25^{-}$ T cells, isolated from healthy donors, were treated with PTEN-specific siRNA (red) or scrambled control siRNA (gray, Ø) using the AMAXA® device. 48 h after transfection, PTEN expression was analyzed by flow cytometry.
